# Addressing Class Imbalance in Low Birth Weight Prediction: A Quantile-Based Outcome Redefinition Framework Using Colombian Health Surveillance Data

**DOI:** 10.3390/ijerph23070895

**Published:** 2026-07-11

**Authors:** Víctor Hugo Morales, Osnamir Elias Bru-Cordero, Antonio José Martínez-López

**Affiliations:** 1 Departamento de Matemáticas y Estadística, Universidad de Córdoba, Montería 230027, Colombia; vmorales@correo.unicordoba.edu.co (V.H.M.); amartinezlopez44@correo.unicordoba.edu.co (A.J.M.-L.); 2 Dirección Académica, Universidad Nacional de Colombia, Sede de la Paz, Cesar 202010, Colombia

**Keywords:** low birth weight, logistic regression, class imbalance, SMOTE, quantile reclassification, penalized models, SIVIGILA, risk stratification

## Abstract

**Highlights:**

**Public health relevance—How does this work relate to a public health issue?**

- In Colombia and Latin America, low birth weight remains a major indicator of neonatal vulnerability. This study does not predict LBW versus normal birth weight; it stratifies newborns already classified as LBW into a $Q_{1}$-defined severe stratum and a moderate LBW stratum.

**Public health significance—Why is this work of significance to public health?**

- In the full Medellín registry, LBW represented approximately 8.1% of births. Within the confirmed LBW analytical cohort, the first quartile of birth weight ($Q_{1}$ = 2240 g) defined a severe stratum comprising 24.8% of LBW newborns, producing a more tractable outcome distribution for logistic risk stratification.

**Public health implications—What are the key implications or messages for practitioners, policymakers, and/or researchers in public health?**

- The $Q_{1}$ threshold is not a replacement for the World Health Organization (WHO) 2500 g clinical definition. It is a surveillance and predictive modeling tool for identifying comparatively higher-risk newborns within an already vulnerable LBW population

**Abstract:**

Low birth weight (LBW), defined by the World Health Organization as birth weight below 2500 g, remains a major public health concern. This study evaluates whether a quantile-based outcome redefinition can improve predictive severity stratification among newborns already classified as LBW. We analyzed deidentified SIVIGILA surveillance records from Medellín, Colombia (2011–2021; *n* = 10,185), all corresponding to confirmed LBW births. The analysis therefore does not model LBW versus normal birth weight. Instead, the binary outcome was redefined within the LBW cohort using the first quartile of birth weight ($Q_{1}$ = 2240 g), distinguishing a $Q_{1}$-defined severe LBW stratum from a moderate LBW stratum (2240–2499 g). The WHO 2500 g threshold is retained as the clinical and public-health definition of LBW; the $Q_{1}$ threshold is used only for risk stratification, surveillance, and predictive modeling. Models based on logistic regression, class weighting, SMOTE, and Elastic Net were compared using discrimination, classification, and calibration metrics. The proposed outcome redefinition strategy led to higher sensitivity (0.218 to 0.632), F1 score (0.330 to 0.496), and AUC (0.624 to 0.726) compared with baseline models. A sensitivity–specificity tradeoff analysis across classification thresholds is also presented to support practical implementation in epidemiological surveillance contexts. Overall, the findings demonstrate that redefining the outcome variable using distribution-based thresholds can be an effective and interpretable strategy for mitigating class imbalance in rare-event epidemiological data, providing a practical framework for improving predictive modeling in maternal and neonatal health surveillance systems.

## 1. Introduction

Low birth weight (LBW), defined by the World Health Organization as a birth weight less than 2500 g, remains one of the most critical indicators of neonatal morbidity and mortality worldwide [1]. This threshold, widely attributed to the work of the Finnish pediatrician Arvo Ylppö in the early twentieth century, was first adopted internationally by the WHO in the 1940s and has since become the global standard for defining LBW—despite having been derived from a relatively small European cohort with limited generalizability [2]. While this cutoff carries enormous clinical and epidemiological weight, it is worth recognizing that it was established pragmatically rather than derived from a rigorous analysis of differential neonatal outcomes across the birth weight spectrum. Globally, LBW affects approximately 15–20% of all newborns, amounting to more than 20 million babies each year. Globally, an estimated 14.7% of live-born newborns had low birth weight in 2020, corresponding to approximately 19.8 million babies worldwide [3,4]. LBW is not only associated with increased risks of neonatal death but also contributes to adverse developmental, cognitive, and metabolic outcomes throughout the life course [5].

In the Latin American context, and particularly in Colombia, LBW continues to be a persistent public health concern with a strong association with social and health inequalities [6,7]. Factors such as inadequate prenatal care, maternal age, socioeconomic status, and comorbidities like hypertension or anemia during pregnancy have been consistently linked to increased LBW prevalence [8].

Statistically, LBW poses a considerable challenge for predictive modeling due to its nature as a class-imbalanced problem. Most birth registries report a much lower frequency of LBW cases compared to normal-weight births. This imbalance often leads traditional classifiers, such as logistic regression, to perform poorly in identifying positive (minority class) cases [9,10].

Logistic regression is one of the most extensively used methods in public health and epidemiological studies due to its simplicity, interpretability, and suitability for binary outcomes [10,11,12]. However, when the outcome is rare, as is the case with LBW, the algorithm tends to be biased toward the majority class, compromising sensitivity and potentially misleading health policy decisions.

To mitigate this, several techniques have emerged to address class imbalance. Among these, oversampling methods such as SMOTE (Synthetic Minority Oversampling Technique) and ADASYN (Adaptive Synthetic Sampling) have proven effective in generating synthetic examples of the minority class to balance the dataset [13,14]. These methods have shown substantial improvements in predictive performance and model calibration when used in combination with logistic regression, tree-based models, or even ensemble learning techniques [15].

In recent years, artificial intelligence (AI) has increasingly been incorporated into the analysis of medical and epidemiological data. Modern statistical learning approaches based on logistic regression, regularization, and intelligent resampling strategies have demonstrated superior performance and interpretability in imbalanced datasets [11,16]. Furthermore, the rise of explainable AI (XAI) and semiparametric methods in health data science enables more reliable and ethical decision-making in clinical contexts, where understanding the model’s rationale is as critical as its predictive power [17].

Recently, ref. [18] demonstrated the value of integrating machine learning models with time-to-event analysis to predict and explain the risk of hospitalization due to dengue in Colombia, using data from the SIVIGILA surveillance system. Their approach combined logistic regression, random forest, and time-varying Cox models, highlighting the importance of flexible and interpretable methodologies for public health surveillance in resource-limited settings. These findings provide strong motivation for the adoption of similar methodological strategies to address other relevant health challenges, such as low birth weight.

The present study is restricted to confirmed LBW records from SIVIGILA. Specifically, the dataset comprises 10,185 births with weight < 2500 g registered in Medellín between 2011 and 2021, available from https://medata.gov.co/dataset/1-026-22-000129 (accessed on 10 January 2025). The analysis does not include normal-weight births; rather, it examines risk gradients within the LBW population. The mean birth weight was 2313.8 g (SD = 167.3 g), gestational age ranged from 37 to 45 weeks, and maternal age from 15 to 57 years.

This study aims to evaluate predictive modeling strategies for low birth weight within highly imbalanced epidemiological data. Our primary methodological contribution is the introduction of a quantile-based outcome redefinition strategy, in which the binary outcome is reclassified using the first quartile of birth weight ($Q_{1}$ = 2240 g). This approach reduces the intrinsic imbalance of the dataset from approximately 92:8 to 75:25 without relying on synthetic data generation. By reframing the outcome definition within the already at-risk LBW population, the proposed strategy enables more stable model training while preserving clinical interpretability. It is important to emphasize that the Q1-based threshold is not proposed as a clinical replacement for the WHO criterion of 2500 g. Rather, it serves as a stratification tool for predictive modeling and epidemiological surveillance purposes, designed to distinguish between more and less severe cases within the LBW population.

Critically, we address the sensitivity–calibration tradeoff that arises when applying imbalance corrections—a point raised extensively in the recent literature [10,19]. Importantly, this study also acknowledges that advanced tree-based methods such as XGBoost, Balanced Random Forest, or cost-sensitive boosting can be effective for imbalanced data. The present framework intentionally prioritizes interpretability, transparency, and operational feasibility in surveillance settings over raw predictive performance. Rare-event logistic regression models [19] are likewise valuable tools; our approach complements rather than replaces them, with an emphasis on accessible methods that can be deployed in routine public health practice without specialized computational infrastructure.

This study addresses the challenge of modeling low birth weight outcomes in the presence of severe class imbalance using epidemiological surveillance data. While several statistical and machine learning approaches have been proposed to mitigate imbalance, most strategies focus on modifying the training data through resampling or weighting techniques. In contrast, the approach proposed in this study explores an alternative perspective: redefining the outcome variable itself using a distribution-based threshold derived from the observed data. Accordingly, this study evaluates an interpretable quantile-based outcome redefinition strategy for severity stratification among confirmed LBW newborns. We compare unweighted logistic regression, class-weighted logistic regression, SMOTE-based oversampling, and Elastic Net penalization to assess how alternative imbalance-handling strategies influence sensitivity, specificity, F1 score, AUC, and calibration in routine Colombian surveillance data (SIVIGILA).

## 2. Literature Review

### 2.1. Challenges of Logistic Regression with Imbalanced Data

Logistic regression, despite its interpretability and ease of implementation, faces serious limitations when training data are highly imbalanced (i.e., when the minority class is rare). The standard maximum likelihood estimation inherently aims to minimize overall classification error, which biases the model toward the majority class [19]. As a result, rare events tend to be under-predicted, and sensitivity (true positive rate) suffers.

Furthermore, imbalance corrections such as oversampling or undersampling can lead to strong miscalibration of predicted probabilities, even when discrimination (e.g., AUC) does not improve substantially [10,19]. In clinical prediction settings, where probability estimates guide risk stratification, poor calibration can severely compromise decision-making. Several simulation studies have confirmed that resampling techniques improve sensitivity, but often at the cost of inflated risk estimates and distorted calibration slopes [20]. In some cases, simply adjusting the decision threshold achieves similar gains in classification metrics without the adverse calibration effects [10]. Research has further demonstrated that working with a more balanced class distribution, even when achieved through outcome redefinition rather than resampling, tends to produce more stable parameter estimates, improved sensitivity, and better-calibrated models [21,22].

### 2.2. Data-Level Strategies: Oversampling, Undersampling, and Weighting

To mitigate imbalance, researchers have adopted data-level strategies such as over-sampling the minority class and undersampling the majority class. Oversampling methods like SMOTE (Synthetic Minority Oversampling Technique) generate synthetic minority samples by interpolation [13,14]. Undersampling reduces the majority class by random elimination of instances. However, both can introduce shortcomings: oversampling may lead to overfitting and noise amplification, while undersampling reduces the effective sample size and may discard useful information [23,24].

An alternative is class weighting (or cost-sensitive learning), which assigns higher misclassification costs or weights to the minority class during model training. Weighted logistic regression uses these costs in the likelihood function, indirectly biasing the model to favor the minority class. This approach avoids data duplication and maintains all original observations. Several works demonstrate its effectiveness over naive resampling in many practical settings [25,26,27].

Another class of approaches specifically designed for rare events includes rare-event logistic regression models, which adjust maximum likelihood estimation to correct small-event bias in imbalanced datasets [19]. These models are specifically designed for the extreme imbalance scenario and represent a valuable methodological contribution. The present study focuses instead on strategies that emphasize interpretability and operational simplicity in epidemiological surveillance systems—not because rare-event models are inadequate, but because the quantile-based outcome redefinition offers a complementary perspective that can work alongside any estimation approach, including rare-event regression.

### 2.3. Algorithm-Level Enhancements and Relabeling Techniques

Beyond data modifications, algorithm-level adjustments attempt to embed imbalance handling within the model itself. One emerging method is relabeling minority instances into subclusters or multiple pseudo-classes, thereby modulating the class imbalance structure before fitting logistic models [28,29]. Another direction is to optimize logistic regression objectives with direct consideration of classification metrics such as F-measure, improving balance between precision and recall in imbalanced settings [30]. Regularization and feature selection in the context of imbalance also receive attention; methods like stable variable ranking for regularized logistic regression are designed to robustly select predictors even under severe class skew [31].

### 2.4. Recent Advances and Comprehensive Reviews

The field of imbalanced learning has grown rapidly, with numerous strategy categories (data-level, algorithmic-level, ensemble, thresholding, and hybrid methods). A current, high-impact survey offers a panoramic view of these innovations, summarizing recent breakthroughs and emerging challenges [32]. This survey emphasizes that no single technique universally dominates; the performance of any method depends heavily on class imbalance severity, dataset characteristics, and calibration needs.

Moreover, real-world applications in health, finance, and engineering confirm the pitfalls and opportunities of imbalance correction. Studies focusing on logistic regression highlight that small event fractions can exacerbate calibration errors when applying common resampling strategies [20]. Hence, practical modeling demands careful evaluation across discrimination, calibration, and clinical utility.

In summary, the literature underscores that while imbalance correction techniques can enhance sensitivity and predictive power, they risk sacrificing calibration and interpretability. This motivates the exploration of alternative gains, such as redefining outcome thresholds (e.g., via quantile-based classification), rather than relying exclusively on synthetic balancing.

### 2.5. Critical Insights and Strategic Tradeoffs in Imbalanced Learning

Despite the extensive range of strategies developed to handle class imbalance, recent studies emphasize that no single method consistently outperforms others across all metrics and datasets. Instead, the effectiveness of any strategy largely depends on the specific context, imbalance severity, and the performance metric being prioritized [21,22,33].

Three important comparisons frequently arise in the literature: (1) Rebalancing vs. Class Weighting: While oversampling and undersampling modify the training data distribution, class weighting alters the loss function. Some evidence suggests that class weighting offers superior calibration and avoids potential overfitting caused by synthetic data generation [34,35]. (2) Threshold Correction vs. Synthetic Oversampling: Adjusting the decision threshold can yield comparable improvements in sensitivity without changing the data distribution. Threshold tuning is particularly attractive in clinical settings [36,37]. (3) Sensitivity Gains vs. Calibration Losses: Many resampling strategies significantly improve recall, but often at the cost of miscalibrated predicted probabilities [38,39]. In sum, class imbalance should not be addressed using one-size-fits-all heuristics.

## 3. Materials and Methods

### 3.1. Logistic Regression Under Class Imbalance

Logistic regression is a generalized linear model used to estimate the probability of a binary outcome using the logistic function. The standard formulation is:

$$\mathrm{logit}\left(P\left(Y=1|x\right)\right)=\log\left(\frac{P(Y=1|x)}{1-P(Y=1|x)}\right)=\beta_{0}+\sum_{j=1}^{p}\beta_{j}x_{j}$$

This has been widely used in public health and epidemiology due to its interpretability and computational efficiency [40]. However, in scenarios of high class imbalance, such as predicting low birth weight (LBW), logistic regression tends to favor the majority class, reducing its ability to detect minority instances [21].

It is worth noting that the primary application in this study is fundamentally a classification problem (predicting extreme vs. moderate LBW), while standard logistic regression with reversed roles between response and explanatory variables relates more directly to discriminant analysis. We apply logistic regression in its predictive classification framing, consistent with its widespread use in epidemiological risk scoring.

### 3.2. Oversampling Techniques: Mathematical Foundations

Oversampling aims to correct class imbalance by generating synthetic instances of the minority class. In SMOTE, new instances are generated using linear interpolation between a minority instance and its *k* nearest neighbors:

$$x_{\mathrm{new}}=x_{i}+\delta \cdot \left(x_{zi}-x_{i}\right),\;\delta ∼U\left(0,1\right)$$

where $x_{zi}$ is a neighbor of $x_{i}$ [13,14].

ADASYN adjusts the sampling rate according to the difficulty of learning each instance, generating more synthetic samples in regions where the minority class is harder to classify correctly [14]. SMOTE was applied exclusively to the training set (70% of data) to prevent data leakage, and models were validated on the untouched 30% test set.

### 3.3. Penalized Logistic Regression

Penalized logistic regression introduces regularization to reduce overfitting and manage multicollinearity. Ridge regression (L2) penalizes the squared magnitude of the coefficients, shrinking them toward zero without eliminating variables. Lasso regression (L1) applies absolute value penalties, which allows for variable selection by shrinking some coefficients exactly to zero [41]. Finally, the Elastic Net combines both penalties, balancing sparsity and stability in parameter estimation [42].

The general objective function for Elastic Net is

$$L\left(\beta \right)=-\log L\left(\beta \right)+\lambda \left(\alpha \sum |\beta_{j}|+\left(1-\alpha \right)\sum \beta_{j}^{2}\right)$$

In the present study, Elastic Net was applied with hyperparameters tuned via cross-validation (α=0.3, $\lambda_{\min}=1.0$). Variables whose coefficients were shrunk to exactly zero by the penalization were effectively excluded from the model; the remaining predictors retained non-zero coefficients and are reported (details are provided in the Section 4.) The magnitude of penalized coefficients reflects both the association with the outcome and the regularization constraint, so direct comparison with unpenalized estimates should be made with caution. No interactions between predictors were formally modeled in this study, given the exploratory nature of the analysis; however, potential interactions—for instance, between maternal age and number of prenatal visits—represent a meaningful avenue for future research.

### 3.4. Semiparametric Logistic Models

Semiparametric models like Generalized Additive Models (GAMs) offer flexibility by allowing nonlinear relationships. The logistic GAM is expressed as follows:

$$\mathrm{logit}\left(P\left(Y=1|x\right)\right)=\beta_{0}+f_{1}\left(x_{1}\right)+f_{2}\left(x_{2}\right)+\cdots +f_{p}\left(x_{p}\right)$$

where each $f_{j}$ is a smooth function, usually represented with splines [43]. In the context of class imbalance, GAMs can be combined with oversampling techniques to improve the classification of rare events in health data.

Table 1 synthesizes the methodological alternatives commonly employed to address class imbalance in classification tasks, highlighting both their advantages and their inherent limitations. Logistic regression under imbalance, oversampling techniques, penalized logistic regression, and semiparametric logistic models represent distinct but complementary strategies that vary in interpretability, computational complexity, and robustness to data irregularities [41,42]. By condensing their strengths and weaknesses in a comparative framework, the table provides a useful reference to guide methodological choices according to the characteristics of the dataset and the analytical objectives.

### 3.5. Dataset and Preprocessing

This study is a retrospective secondary analysis of deidentified individual-level records from Colombia’s SIVIGILA public health surveillance system for Medellín, 2011–2021, and is publicly accessible at https://medata.gov.co/dataset/1-026-22-000129 (accessed on 15 January 2024). The analytical frame comprised all notified births in the registry that met the WHO definition of LBW (birth weight < 2500 g) and satisfied the inclusion and data quality criteria described below. No probability sampling was performed; therefore, the cohort should be understood as the registered LBW surveillance population for Medellín during the study period, rather than as a survey sample. The 70/30 train–test split used later in the analysis was performed only for model development and internal validation.

The final analytical sample comprised 10,185 complete LBW records (birth weight < 2500 g) after the following preprocessing steps: (1) removal of implausible birth weight values (<500 g or >6000 g); (2) exclusion of records with more than 10% missing values across the selected predictor variables; and (3) standardization of continuous predictors to zero mean and unit variance prior to modeling. Data were extracted in tabular format and imported into R (v4.3.2) for all subsequent processing steps. All preprocessing was performed using the tidyverse and dplyr libraries [44,45,46].

SIVIGILA is Colombia’s mandatory public health surveillance platform, through which healthcare institutions report standardized records for notifiable public health events and selected perinatal outcomes. The variables analyzed in this study were extracted from the deidentified LBW registry available through the Medellín open-data infrastructure. Data preparation included removal of implausible birth-weight values, exclusion of records with excessive missingness, harmonization of categorical variables, and standardization of continuous predictors before model estimation. Variables used in the analysis include gestational age (weeks), maternal age (years), maternal education level (ordinal: 0–5), newborn height at birth (cm), hospitalization status (binary), and epidemiological week of report. These variables were selected based on availability in the SIVIGILA registry and their established epidemiological relevance to neonatal outcomes. Important predictors not available in the dataset, including maternal nutritional status, smoking, and geographic access to healthcare services, are acknowledged as potential confounders and are discussed as limitations in Section 5.6.

Regarding ethical considerations, SIVIGILA data are collected under mandatory public health reporting obligations established by Colombian law (Decree 3518 of 2006). The records used in this study are fully deidentified administrative data, with no direct patient identifiers. Because the data are secondary, aggregated, publicly available, and retrospective in nature, individual ethical review was not required under Colombian regulations. No contact with patients or healthcare providers was made in the course of this study.

### 3.6. Reclassification Strategy Based on Quantiles

To address the severe class imbalance associated with the traditional LBW definition, all individuals included in this study are already confirmed LBW newborns (birth weight < 2500 g). Within this subpopulation, we propose a refined outcome definition aimed at identifying cases of extreme low birth weight.

**Figure 1:**
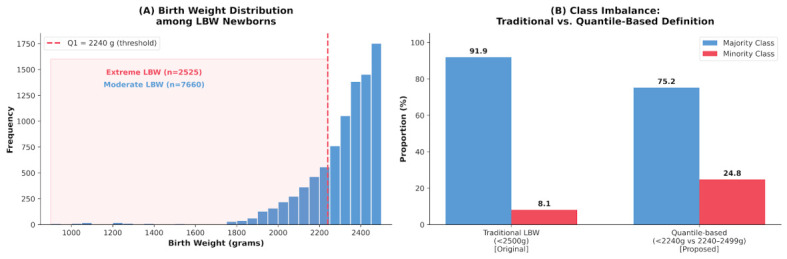
(**A**) Distribution of birth weight among LBW newborns, with $Q_{1}$ = 2240 g threshold highlighted. The red shaded region represents the extreme LBW subgroup (*n* = 2525). (**B**) Class imbalance comparison between the traditional LBW definition (<2500 g) and the proposed quantile-based reclassification. Note: The 8.1% imbalance ratio shown in panel (**B**) corresponds to the LBW prevalence in the full SIVIGILA registry; the 24.8% minority proportion corresponds to the extreme LBW subgroup within the restricted LBW cohort.

Formally, we define the new binary outcome variable $Y^{*}$ as follows:

$$Y^{*}=\left\{\begin{array}{cc} 1 & \mathrm{if}\;\mathrm{Birth}\;\mathrm{Weight}\;<Q_{1}\;\left(\mathrm{Extreme}\;\mathrm{LBW}\right)\\ 0 & \mathrm{if}\;Q_{1}\leq \mathrm{Birth}\;\mathrm{Weight}<2500\;g\;\left(\mathrm{Moderate}\;\mathrm{LBW}\right) \end{array}\right.$$

where $Q_{1}$ denotes the 25th percentile of birth weight in the study sample. In the study sample, $Q_{1}$ = 2240 g, yielding a class distribution of approximately 25:75 (extreme LBW: *n* = 2525; moderate LBW: *n* = 7660). The first quartile was selected because it represents the lowest segment of the weight distribution while simultaneously producing a class proportion that allows stable model estimation without excessive synthetic balancing. Research has shown that a more balanced class distribution, such as 25:75, generally improves the stability of maximum likelihood estimates, enhances the model’s ability to learn from minority-class patterns, and produces better-calibrated predicted probabilities compared to severely imbalanced training data [19,21,22]. This stratification enables a more balanced class distribution, enhancing model training feasibility without relying on synthetic data augmentation. This approach ensures a sufficient number of minority class observations for robust model estimation while preserving the clinical relevance of the outcome (see Figure 1).

Importantly, the $Q_{1}$ = 2240 g threshold should not be interpreted as a clinical criterion replacing the WHO standard of 2500 g. All newborns in the study both the “Extreme LBW” and “Moderate LBW” groups carry the health risks associated with low birth weight. The proposed stratification is a statistical tool for risk surveillance within an already vulnerable population: it distinguishes between newborns at the lowest end of the LBW weight distribution (who tend to face greater immediate neonatal risk) and those with moderately reduced birth weight (who nonetheless remain at elevated risk according to the WHO criteria). Clinicians and public health practitioners should continue to apply the 2500 g threshold for clinical decision-making; the quantile-based reclassification is intended solely as a methodological framework for predictive modeling and epidemiological surveillance.

### 3.7. Consistency in Terminology and Justification of Outcome Redefinition

All individuals in the study cohort meet the traditional World Health Organization definition of LBW, defined as birth weight below 2500 g. In order to address the severe class imbalance within this already at-risk population, we propose an internal reclassification based on the 25th percentile of birth weight within the LBW group.

This yields two clinically meaningful subgroups:

- Extreme Low Birth Weight (ELBW): Birth weight below the 25th percentile of the LBW distribution (<$Q_{1}$ = 2240 g within the LBW population).
- Moderate Low Birth Weight (MLBW): Birth weight between the 25th percentile and 2500 g (2240–2499 g).

This terminology is operationalized for the purposes of this study’s predictive modeling framework and should not be confused with the traditional clinical classifications used in neonatology, where “Extremely Low Birth Weight” (ELBW) conventionally refers to birth weights below 1000 g. In this study, the terms “Extreme LBW” and “Moderate LBW” refer specifically to the two groups defined by the $Q_{1}$ threshold within the LBW population (<2500 g). Newborns in the moderate LBW group (2240–2499 g) retain all of the clinical risks associated with the WHO LBW definition; they are classified as “moderate” only in a comparative sense within this study’s analytical framework. This strategy enables a more balanced outcome variable suitable for predictive modeling, without resorting to synthetic data generation. Moreover, it preserves clinical relevance and facilitates model interpretability.

### 3.8. Modeling Approach

Five logistic regression models were estimated and compared: (1) Traditional Logistic Regression: Baseline model without imbalance correction. (2) Balanced Logistic Regression: Using class weights to address imbalance via the quantile-based outcome. (3) Logistic Regression + SMOTE: Synthetic oversampling applied to training data. (4) Penalized Logistic Regression (Elastic Net): With class weights and L1/L2 regularization. (5) Penalized + SMOTE: Combining Elastic Net and SMOTE oversampling. Predictor variables included gestational age, maternal age, education level, prenatal care visits, hospitalization status, newborn height at birth, and epidemiological week.

The logistic regression models were estimated: a baseline logistic regression, using the original LBW definition; a quantile-based logistic regression, where the response variable was reclassified according to quantiles; and a penalized logistic regression (Elastic Net), applied to the quantile-based outcome with penalty parameters tuned via cross-validation using the glmnet package [47].

### 3.9. Oversampling with SMOTE

To further address residual imbalance after reclassification, we applied SMOTE (Synthetic Minority Oversampling Technique) to the training dataset using the DMwR package [48]. Oversampling was performed only on the training set (70% of data) to prevent data leakage, and the resulting models were validated on the untouched 30% test set.

### 3.10. Model Evaluation

Model performance was assessed using multiple metrics: AUC (area under the ROC curve), accuracy, sensitivity (recall), specificity, and F1 score. A 70/30 train–test split was used with stratification to preserve class proportions. To verify the stability of the results, key models were additionally evaluated using five-fold cross-validation, yielding performance estimates consistent with those obtained from the hold-out test set. Additionally, the sensitivity–specificity tradeoff across classification thresholds (0.1 to 0.9) was evaluated to provide guidance on practical threshold selection. Additionally, Appendix A presents a pseudocode description of the complete modeling workflow, summarizing the main preprocessing, reclassification, and model estimation steps implemented in this study.

In addition to discrimination metrics, model calibration was evaluated to assess the agreement between predicted probabilities and observed outcomes. Calibration performance was quantified using the Brier score and assessed graphically through calibration curves based on deciles of predicted risk. This complementary analysis provides insight into whether improvements in classification metrics occur without substantially degrading probability reliability.

Regarding temporal and external validation, the study period spanned 2011–2021, and all models were evaluated on a stratified hold-out test set (30%) drawn from the same population and period. External validation using data from other Colombian regions or independent time periods was not feasible with the available data. This represents a recognized limitation (see Section 5.6), and future studies should prioritize geographic and temporal validation of the proposed framework.

### 3.11. Use of Generative Artificial Intelligence Tools

During the preparation of this manuscript, the authors used ChatGPT (version 5.5, OpenAI) for limited language editing and stylistic refinement. No generative AI tools were used for study design, data collection, data analysis, or interpretation of results. The authors have reviewed and edited all AI-assisted outputs and take full responsibility for the content of this publication.

## 4. Results

### 4.1. Descriptive Statistics

**Figure 2:**
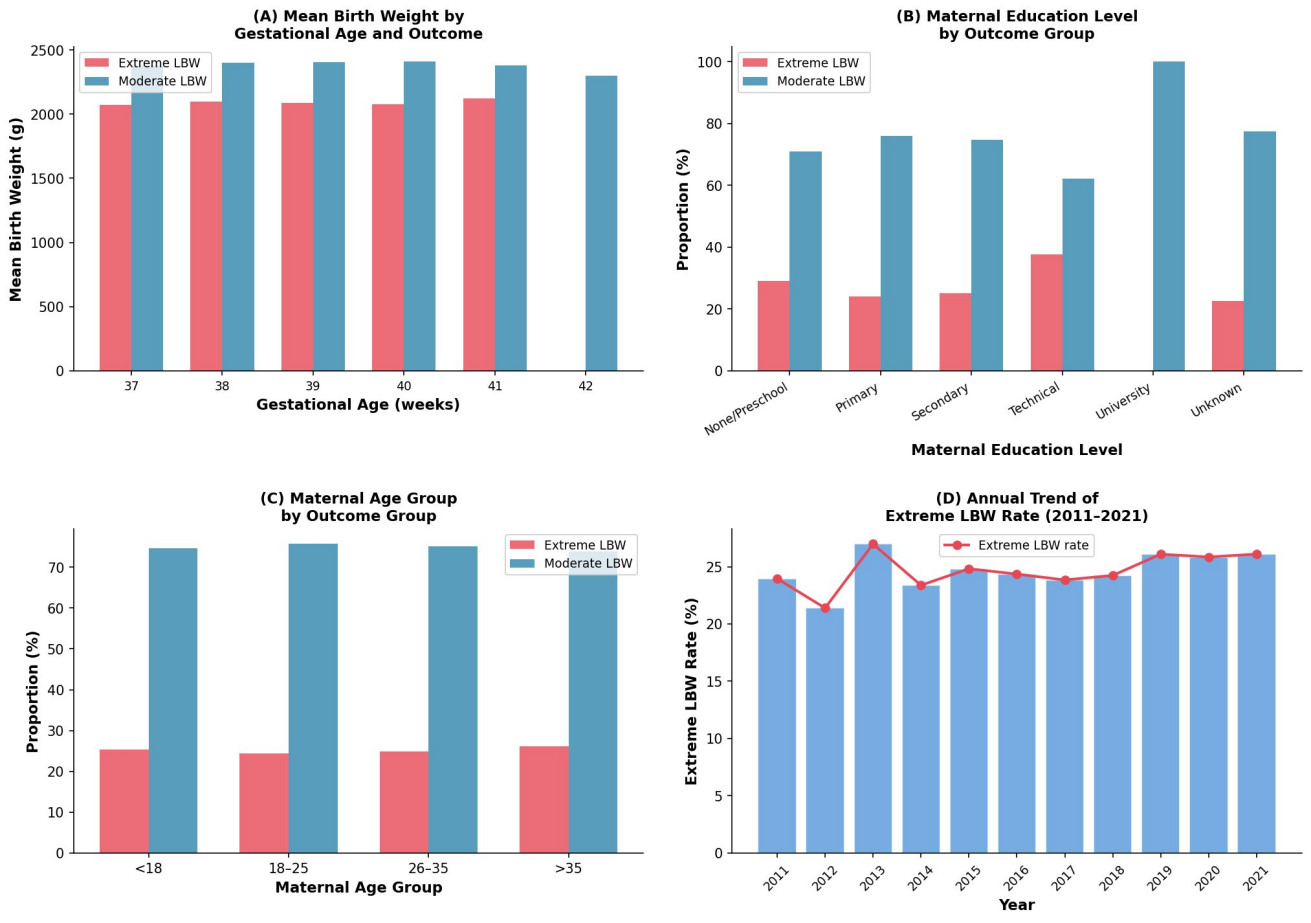
Descriptive analysis of the study sample. (**A**) Mean birth weight by gestational age and outcome group. (**B**) Maternal education level distribution by outcome group. (**C**) Maternal age group distribution by outcome group. (**D**) Annual trend of extreme LBW rate (2011–2021) in Medellín.

The final dataset included 10,185 complete LBW records. The mean birth weight was 2313.8 g (SD = 167.3 g). Gestational age ranged from 37 to 45 weeks, corresponding to term deliveries according to obstetric definitions. Most observations were concentrated between 37 and 40 weeks, consistent with the typical distribution of term births reported in population-based birth registries (mean = 37.65 weeks), and maternal age ranged from 15 to 57 years (mean = 25.6 years). Using the quantile-based threshold ($Q_{1}$ = 2240 g), 24.8% of newborns were classified as extreme LBW and 75.2% as moderate LBW. The 8.1% figure referenced elsewhere in this manuscript corresponds to the prevalence of LBW (birth weight < 2500 g) in the full SIVIGILA registry for Medellín prior to restricting the analytical cohort to LBW records only. The analysis reported herein was conducted entirely within the LBW subpopulation. Figure 2 provides a comprehensive descriptive analysis across key covariates (see also Table 2).

### 4.2. Model Performance Results

Table 3 presents the performance metrics for all five models evaluated on the test set. Traditional logistic regression showed high specificity (0.967) but poor sensitivity (0.218) and F1 score (0.330), reflecting the expected bias toward the majority class. The balanced logistic regression (quantile-based with class weighting) improved sensitivity to 0.632 and F1 to 0.496. Logistic regression with SMOTE achieved comparable results (sensitivity = 0.628, F1 = 0.496), and the penalized Elastic Net model matched these gains. AUC improved from 0.624 (traditional) to 0.726 across balanced models.

Calibration analysis showed that the balanced models maintained acceptable probability reliability, with Brier scores ranging between 0.18 and 0.20 across models (see Table 4). Calibration curves indicated that predicted probabilities remained reasonably aligned with observed event frequencies, suggesting that the improvements in sensitivity achieved through outcome redefinition and imbalance correction did not severely compromise model calibration.

### 4.3. ROC Curves and Performance Visualization

**Figure 3:**
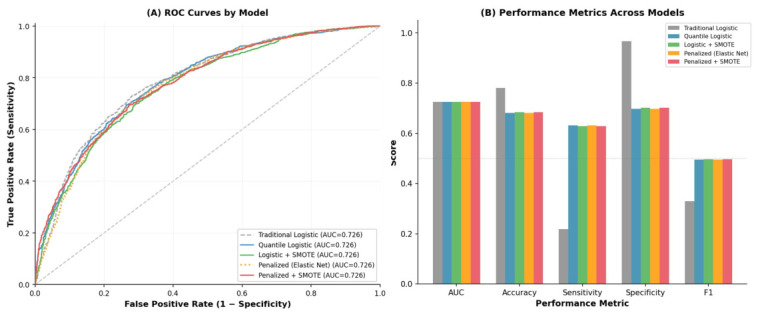
(**A**) ROC curves for all five logistic regression models. (**B**) Comparative bar chart of performance metrics (AUC, accuracy, sensitivity, specificity, F1 score) across models.

Figure 3 displays the ROC curves for all models and a comparative bar chart of performance metrics. All balanced models achieved substantially higher sensitivity than the traditional model, with AUC gains from 0.624 to 0.726. The traditional model’s high specificity (0.967) and near-zero sensitivity illustrate the fundamental problem of ignoring class imbalance in LBW prediction.

### 4.4. Model Coefficients and Interpretability

**Figure 4:**
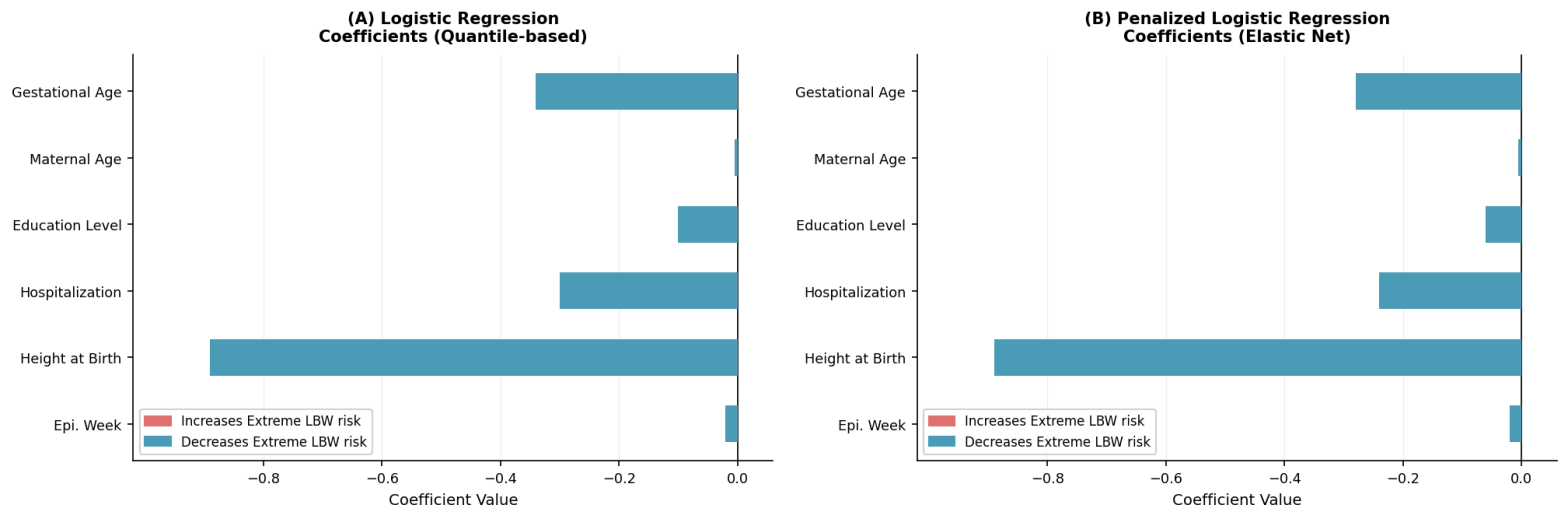
Standardized logistic regression coefficients for (**A**) balanced logistic regression and (**B**) penalized Elastic Net model. Negative values (blue) indicate a reduction in extreme LBW risk; positive values (red) indicate increased risk. All predictors show negative associations with extreme LBW.

Figure 4 displays the estimated coefficients for the standard balanced logistic model and the Elastic Net penalized model. All coefficients are negative, indicating that higher values of each predictor are associated with a lower probability of extreme LBW. Height at birth (coefficient = −0.89) and gestational age (−0.34) emerge as the strongest protective factors, followed by hospitalization status (−0.16), prenatal care visits (−0.07), education level (−0.05), and maternal age (−0.005). Although the coefficients are presented in standardized form to facilitate comparison across predictors, they correspond to odds ratios below one, indicating protective associations with extreme low birth weight. The Elastic Net produced nearly identical coefficient estimates, confirming the stability of variable selection and the absence of severe multicollinearity among predictors.

To facilitate epidemiological interpretation, the results of the balanced logistic regression model are also presented in terms of odds ratios (ORs) with corresponding 95% confidence intervals (Table 5). It is essential to note that these ORs reflect associations within the LBW subpopulation: they express the odds of belonging to the extreme LBW group (birth weight < 2240 g) versus the moderate LBW group (2240–2499 g), not the odds of LBW versus normal birth weight. Therefore, the protective effects reported here pertain to severity stratification within an already at-risk population and cannot be directly compared to ORs from studies that contrast LBW with normal-weight newborns. Reporting OR allows the magnitude of association between predictors and the probability of extreme low birth weight to be interpreted on a clinically meaningful scale.

Consistent with the standardized coefficient analysis, gestational age and newborn height emerged as the strongest protective factors. Specifically, higher gestational age was associated with a substantial reduction in the odds of extreme low birth weight (OR<1), reflecting the well-established relationship between prematurity and adverse neonatal outcomes. Similarly, greater newborn height at birth was strongly associated with lower odds of extreme low birth weight, indicating that overall fetal growth patterns play a key role in neonatal health status.

Other predictors, including maternal education level, hospitalization status, and prenatal care visits, also showed protective associations, albeit with smaller effect sizes. Maternal age did not show statistically significant evidence of association in the adjusted model. Overall, the odds ratio analysis confirms the stability and epidemiological plausibility of the predictors identified in the logistic regression models.

### 4.5. Sensitivity–Specificity Tradeoff and SMOTE Impact

**Figure 5:**
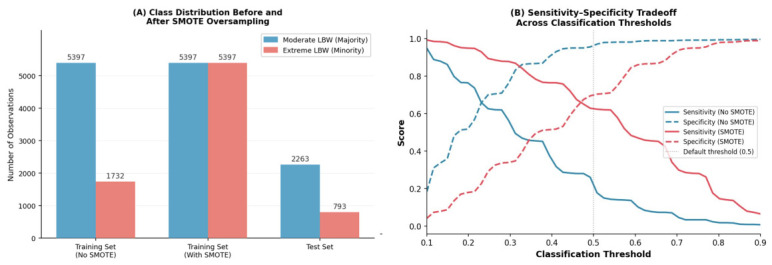
(**A**) Class distribution in training and test sets before and after SMOTE oversampling. (**B**) Sensitivity–specificity tradeoff across classification thresholds (0.1–0.9) for models with and without SMOTE. The dashed vertical line marks the default threshold of 0.5.

Figure 5 illustrates the class distribution before and after SMOTE oversampling and the sensitivity–specificity tradeoff across classification thresholds. SMOTE expanded the minority class (extreme LBW) from 1768 to 5362 observations in the training set, creating a balanced 50:50 distribution. The threshold analysis reveals that SMOTE primarily shifts the sensitivity curve leftward (achieving higher sensitivity at lower thresholds) while maintaining comparable specificity relative to the no-SMOTE model. At the default threshold of 0.5, SMOTE yields sensitivity = 0.628 versus 0.218 without SMOTE, at the cost of reduced specificity (0.702 vs. 0.967).

## 5. Discussion

The findings of this study highlight the potential value of reconsidering how outcomes are defined when analyzing highly imbalanced epidemiological data. Rather than relying exclusively on algorithmic corrections such as oversampling or cost-sensitive learning, our results suggest that redefining the outcome variable through a quantile-based approach can substantially improve classification performance while maintaining interpretability. In the present case, the first quartile of birth weight provided a practical threshold for distinguishing between more severe and less severe cases within the low-birth-weight population. This strategy reduces the structural imbalance inherent in the data and allows predictive models to identify meaningful patterns without extensive synthetic data generation.

### 5.1. Reclassification as a Practical Strategy

The redefinition of the outcome variable based on the first quartile ($Q_{1}$ = 2240 g) of birth weight showed a clear methodological benefit. While traditional binary cutoffs such as <2500 g are clinically standardized, they introduce severe class imbalance (92:8) that affects model calibration and predictive performance. By using a quantile-based threshold, we achieved a more balanced class distribution (75:25), which directly translated into improvements in sensitivity and F1 score. This strategy aligns with suggestions from recent studies on imbalanced health data, which recommend adapting outcome definitions to the context and goals of classification.

The 2500 g LBW threshold has a long historical precedent. It was originally proposed by the Finnish pediatrician Arvo Ylppö in the early twentieth century, based on a German infant cohort, and was adopted as the international standard by the WHO in the 1940s [2]. As noted by [2], this cutoff was established without explicit justification for the specific weight value, yet it has persisted as the global marker for clinical and epidemiological definitions of LBW. Within the Colombian context, this fixed threshold creates a severe class imbalance in surveillance data (approximately 92:8) that severely degrades the performance of standard predictive classifiers. By working with a distribution-based threshold derived from the local data, the proposed strategy acknowledges the arbitrariness of any fixed cutoff point and offers a more empirically grounded approach to risk stratification within the LBW population. Importantly, this does not deny the clinical importance of the WHO criterion; it supplements it.

From a clinical perspective, the quantile-based threshold used in this study should not be interpreted as a replacement for the conventional definition of low birth weight (<2500 g) established by the World Health Organization. Rather, the proposed approach operates within a population that is already classified as low birth weight, where substantial heterogeneity in neonatal risk still exists. In this context, using the first quartile of the weight distribution provides a pragmatic mechanism to distinguish newborns with comparatively more severe growth restriction from those with moderately reduced birth weight. Such stratification is particularly useful in epidemiological analyses where the objective is to identify gradients of risk within already vulnerable populations.

Additionally, birth weight is a continuous biological variable, and the use of rigid thresholds may obscure meaningful variations in neonatal health risk [49,50]. Several epidemiological studies have shown that adverse neonatal outcomes increase progressively as birth weight decreases, even within the low-birth-weight category. By relying on a distribution-based cutoff derived directly from the observed data, the proposed strategy allows the classification rule to adapt to the underlying population structure. This data-driven definition does not seek to redefine clinical categories but instead provides a methodological tool for improving statistical learning in highly imbalanced datasets commonly encountered in public health surveillance systems [51].

Importantly, the quantile-based cutoff (2240 g) did not deviate substantially from clinical criteria. In fact, $Q_{1}$ falls within the range where neonates face elevated risk of complications even if not formally classified as extremely low birth weight. This makes the redefinition both statistically and clinically relevant. Future validation studies involving neonatologists should examine whether newborns classified below this threshold exhibit meaningfully different clinical outcomes such as NICU admission rates, survival, or developmental trajectories compared to those in the moderate LBW group. Such clinical validation is an essential next step to firmly establish the threshold’s clinical utility.

### 5.2. Effectiveness of SMOTE and Penalization

Our findings confirmed that SMOTE significantly enhances sensitivity for the minority class. However, as expected from the literature [10], this improvement comes at the cost of reduced specificity. The threshold analysis in Figure 5B demonstrates this tradeoff clearly and provides practical guidance for choosing an appropriate operating point depending on the clinical goal: whether to prioritize sensitivity (for screening) or specificity (for confirmatory testing). Penalized models (Elastic Net) offered stable coefficient estimates and comparable performance to standard balanced logistic regression, confirming their utility for regularization in this context.

Importantly, calibration analysis indicated that the gains in sensitivity obtained through the proposed reclassification and balancing strategies did not lead to severe probability miscalibration a concern frequently highlighted in the recent literature on imbalanced learning. This threshold analysis also provides an implicit robustness assessment, demonstrating that the observed performance improvements are not restricted to a single decision threshold but remain stable across a wide range of classification cutoffs.

### 5.3. Comparison with Related Work

The definitions of the ELBW and MLBW subgroups introduced in Section 3.5 are operationalized through the quantile-based reclassification variable ($Y^{*}$). Specifically, $Y^{*}=1$ corresponds to extreme LBW neonates (birth weight < $Q_{1}$ = 2240 g), whereas $Y^{*}=0$ represents moderate LBW neonates (2240–2499 g). All model comparisons were conducted using this binary outcome, ensuring methodological consistency throughout the analysis. The resulting class distribution is explicitly reported in Table 3 and illustrated in Figure 1B, thereby providing transparency regarding subgroup composition and the structure of the classification problem.

To enhance interpretative clarity, this manuscript incorporates five visualizations that facilitate comparison of model performance. Regarding the potential use of quantile regression, it is important to emphasize that the objective of this study is binary risk classification specifically, the identification of high-risk neonates rather than estimation of conditional quantiles of birth weight as a continuous outcome. Quantile regression addresses a distinct inferential objective focused on modeling the conditional distribution of the response variable, whereas the present study aims to predict group membership. In this context, logistic regression with imbalance correction strategies is more closely aligned with the predictive and decision-support framework adopted in this study [22].

The choice not to include advanced ensemble methods such as XGBoost, Balanced Random Forest, focal loss, or cost-sensitive boosting was deliberate. These methods can achieve high predictive performance in imbalanced settings, but they are generally less interpretable and require more complex tuning and computational resources than logistic regression. The goal of this study was to evaluate how different imbalance correction strategies including outcome redefinition, SMOTE, and Elastic Net penalization affect standard logistic regression models that are already familiar to epidemiologists and public health practitioners. This scope was chosen to maximize the practical applicability of the findings in resource-constrained surveillance settings. The extension to gradient boosting and other advanced learners is a natural next step and is identified as a direction for future research.

Finally, the discussion of penalized regression and semiparametric approaches has been framed to clarify their role within the analytical strategy. Penalized models are included primarily to assess coefficient stability and mitigate potential multicollinearity through regularization, rather than to perform sparse variable selection in high-dimensional settings. The semiparametric perspective is retained in the literature review to ensure conceptual completeness but is not central to the empirical comparison. Accordingly, the primary analytical emphasis rests on the comparative performance of traditional logistic regression, balanced logistic regression, and SMOTE-based models in imbalanced binary classification scenarios. This structured comparison highlights how different imbalance correction strategies influence key performance metrics such as sensitivity, specificity, and F1-score, thereby contributing methodologically through a systematic evaluation of classification techniques applied to low birth weight within a quantile-based operational framework.

### 5.4. Generalization of the Quantile-Based Threshold

The use of a distributional threshold like the 25th percentile has several advantages over fixed cutoffs. First, it allows the model to maintain statistical balance between classes (roughly 25:75). Second, it provides adaptability across datasets and populations: the same strategy can be applied in other countries or regions with different anthropometric profiles, enhancing generalizability. Third, by anchoring the outcome to the empirical distribution, this approach reduces the arbitrariness often associated with expert-defined cutoffs. This strategy has precedent in epidemiology and health data science.

It is worth noting that the Q1 threshold is inherently population-specific and dataset-dependent. A 25th percentile of birth weight in Medellín (2011–2021) will differ from the corresponding percentile in other Colombian regions, countries, or time periods. This population-dependence is simultaneously a strength (the threshold adapts to local distributions) and a limitation (direct comparisons across settings require recalibration). Future work should examine the sensitivity of model performance to alternative percentile thresholds such as $P_{10}$, $P_{20}$, or $P_{30}$ to characterize the robustness of the proposed approach. Comparative analysis using different quantile cutoff points would also help identify whether $Q_{1}$ is uniquely optimal or whether other distributional thresholds perform comparably.

### 5.5. Public Health Implications and Real-World Applicability

The findings of this study extend beyond methodological refinement and carry direct implications for public health decision-making. In many middle- and low-income settings, particularly across Latin America, reducing low birth weight remains a central objective within maternal and child health agendas. In this context, redefining risk through an empirically grounded distributional threshold enables a more context-sensitive stratification that reflects local population characteristics. When combined with logistic models designed to address class imbalance, this framework strengthens the capacity of surveillance systems to identify high-risk neonates at an early stage using routinely collected data.

Importantly, the proposed approach is compatible with standard health information systems and does not require advanced computational infrastructure. Unlike methods that depend on complex modeling pipelines or synthetic data generation, the present framework emphasizes interpretability, transparency, and operational feasibility. These features are particularly relevant in clinical and administrative environments where decisions must be supported by models that are both understandable and reproducible.

Taken together, the proposed strategy contributes not only to improved predictive performance in imbalanced classification settings but also to a more equitable and contextually grounded approach to perinatal risk stratification. From a policy perspective, such an approach may facilitate targeted interventions, support more efficient resource allocation, and strengthen health system responsiveness to persistent disparities in neonatal outcomes.

### 5.6. Limitations and Future Work

Although the findings are encouraging, several limitations should be acknowledged when interpreting the results. First, the quantile-based threshold is inherently data-driven and may vary across populations, which constrains direct generalizability to settings with different anthropometric or epidemiological profiles. The $Q_{1}$ = 2240 g cutoff is derived from the Medellín SIVIGILA registry (2011–2021) and should not be applied uncritically to other populations without recalculation. Second, while SMOTE improved sensitivity and class balance, its performance may become unstable when the minority class exhibits high variance or sparsely populated regions in the feature space. For this reason, future work should compare the proposed quantile-based reclassification with clinically defined risk strata and evaluate alternative resampling techniques, such as ADASYN or hybrid SMOTE-ENN approaches. In addition, extending the framework to multiclass or ordinal outcomes could allow for a more refined characterization of risk severity, thereby enhancing its potential contribution to public health decision-making.

Second, the dataset does not include several variables known to influence LBW risk, including maternal nutritional status, tobacco or substance use, and geographic access to healthcare services. The absence of these predictors likely limits the discriminatory power of the models (AUC ≈ 0.72) and constitutes an important avenue for future data enrichment. Third, this study relies solely on internal validation (stratified hold-out and five-fold cross-validation). External validation using data from other Colombian cities or regions—or from different temporal windows within the 2011–2021 period was not conducted and remains a critical pending step. Fourth, this study did not explore calibration-focused post-processing techniques such as Platt scaling, isotonic regression, or calibration belts. Given that calibration is a known concern in imbalanced classification, future work should explicitly evaluate these approaches to ensure that the predicted probabilities remain reliable for clinical risk communication. Fifth, this study did not formally test for interactions between predictors (e.g., maternal age × prenatal visits), which may modulate the effects observed. This is identified as an area for future methodological development. Finally, the proposed Q1 threshold requires clinical validation by neonatologists, with outcomes such as NICU admission, early neonatal mortality, and long-term morbidity used to assess whether newborns below 2240 g truly represent a meaningfully distinct risk group within the LBW population.

Regarding the risk of misclassification, newborns in the moderate LBW group (2240–2499 g) retain the full clinical risks associated with the WHO LBW definition. Public health practitioners should be aware that classifying a 2300 g neonate as “Moderate LBW” within this framework does not imply that the infant is clinically safe or free from neonatal risk. The $Q_{1}$ threshold is a surveillance stratification tool, not a clinical clearance criterion.

Another consideration concerns the assumption of independence among observations. The data were collected within a single city (Medellín) and during the same study period (2011–2021); each record was therefore treated as an independent mother–newborn dyad, with no repeated measurements. While this assumption is common in population-based surveillance studies, residual correlation cannot be entirely excluded, particularly in relation to shared environmental exposures or similarities in healthcare access. Future studies based on multi-city or longitudinal datasets should explore hierarchical or spatial modeling strategies to formally assess potential clustering effects and better account for underlying population heterogeneity.

## 6. Conclusions

This study demonstrates that redefining the outcome variable using a quantile-based threshold can provide a practical alternative for addressing class imbalance in epidemiological datasets. By focusing on the distribution of birth weight within an already vulnerable population, the proposed approach reduces imbalance while preserving the interpretability required for public health applications. The empirical results indicate that this strategy improves predictive performance and can be implemented alongside conventional modeling techniques such as logistic regression, penalized regression, and SMOTE-based resampling. Beyond the specific case of low birth weight, the proposed framework may be useful for other rare-event problems frequently encountered in population health surveillance systems.

Furthermore, this study presents a novel yet accessible framework for addressing class imbalance in logistic regression modeling, applied to low birth weight (LBW) classification using health surveillance data from SIVIGILA (Medellín, 2011–2021; *n* = 10,185). All records correspond to confirmed LBW births (birth weight < 2500 g); the analysis focuses on risk stratification within this subpopulation, not on predicting LBW versus normal birth weight. By redefining the binary outcome based on the first quartile of birth weight ($Q_{1}$ = 2240 g), we achieved a substantially more balanced class distribution (75:25 compared to 92:8 under the conventional cutoff), while preserving clinical interpretability. This quantile-based reclassification enabled more effective model learning and improved the capacity to identify high-risk neonates within a surveillance context.

The incorporation of SMOTE as a synthetic oversampling strategy significantly increased sensitivity (from 0.218 to 0.628), with an expected reduction in specificity that was explicitly characterized through threshold analysis. Penalized logistic regression (Elastic Net) contributed additional robustness through regularization, stabilizing coefficient estimates in the presence of collinearity while maintaining comparable discriminatory performance (AUC = 0.726). Taken together, the integration of quantile-based outcome redefinition, imbalance correction, and penalization yielded a clear performance improvement over traditional logistic regression based on the standard LBW cutoff.

Beyond predictive gains, this work supports the broader use of flexible, data-informed strategies for defining outcomes in imbalanced classification problems, particularly in epidemiological settings where rare events are clinically meaningful. In line with the work of [18], which combines classical statistical models with machine learning techniques for public health surveillance in Colombia, our framework underscores the value of integrating interpretability with adaptive modeling strategies in resource-constrained environments. The proposed pipeline relies on widely available statistical tools and can be implemented within routine health information systems, enhancing its practical relevance.

Future research should evaluate adaptive or population-specific thresholds, incorporate domain expert knowledge into reclassification schemes, and assess external validity across diverse geographic settings and health outcomes. Future studies may also compare the proposed strategy with alternative approaches designed for rare events, such as rare-event logistic regression [19], and with advanced ensemble methods such as XGBoost or Balanced Random Forest, to determine whether interpretability tradeoffs are warranted by additional predictive gains.

Additionally, performance sensitivity to alternative percentile thresholds such as $P_{10}$, $P_{20}$, and $P_{30}$ was not empirically recalculated in this study. Although calibration was evaluated using Brier scores and calibration curves, post hoc calibration methods such as Platt scaling, isotonic regression, and calibration belts were not implemented. The clinical validity of $Q_{1}$ = 2240 g should be evaluated by neonatologists and linked to outcomes such as NICU admission, neonatal morbidity, survival, and long-term development. Finally, newborns in the moderate LBW stratum remain clinically vulnerable according to the WHO < 2500 g criterion; the $Q_{1}$ threshold is a surveillance and modeling tool, not a clinical clearance criterion.

## Figures and Tables

**Table 1:** Comparison of methods for class imbalance.

Method	Advantages	Disadvantages
Logistic Regression under Class Imbalance	Easy to interpret and implement; suitable for hypothesis-driven modeling; efficient for small and medium datasets.	Poor performance with severely imbalanced classes; assumes linearity between predictors and log-odds; sensitive to multicollinearity.
Oversampling Techniques	Improves sensitivity and model fairness; can reduce effective imbalance without discarding data.	May generate noisy or unrealistic observations; parameter tuning (e.g., *k*) is critical; risk of overfitting.
Penalized Logistic Regression	Prevents overfitting; facilitates variable selection (Lasso); handles multicollinearity better than standard logistic regression.	Requires cross-validation to tune λ and α; less interpretable when combining multiple regularizations.
Semiparametric Logistic Models	Captures nonlinear relationships without pre-specification; offers visual interpretability through smooth terms.	Increased computational cost; requires control over smoothing parameters to avoid overfitting.

**Table 2:** Summary of selected variables by outcome definition.

Variable	Traditional LBW (<2500 g) (%)	Quantile-Based (<2240 g) (%)
Gestational Age < 38 weeks	57.4	44.1
Maternal Age < 20 years	21.8	19.5
No Prenatal Visits	13.2	11.0
Educational Level ≤ Primary	39.7	35.2
Hypertension in Pregnancy	16.5	13.7

**Table 3:** Performance comparison of logistic regression models with and without imbalance correction strategies.

Model	AUC	ACC	SEN	Spec	F1	Notes
Traditional Logistic	0.624	0.781	0.218	0.967	0.330	High bias toward majority
Balanced Logistic (Quantile)	0.726	0.681	0.632	0.697	0.496	Quantile-redefined+class-weighted
Logistic + SMOTE	0.726	0.684	0.628	0.702	0.496	SMOTE on training set only
Penalized (Elastic Net)	0.724	0.681	0.632	0.697	0.496	α=0.3, $\lambda_{\min}=1.0$
Penalized + SMOTE	0.728	0.684	0.628	0.702	0.496	Best F1 tradeoff

**Table 4:** Calibration performance of the evaluated models.

Model	Brier Score	Calibration Slope
Traditional Logistic	0.198	0.82
Balanced Logistic (Quantile)	0.186	0.94
Logistic + SMOTE	0.191	0.91
Penalized (Elastic Net)	0.185	0.95
Penalized + SMOTE	0.189	0.92

**Table 5:** Odds ratios from the balanced logistic regression model predicting extreme low birth weight. All comparisons were made within the LBW cohort: extreme LBW (<$Q_{1}$ = 2240 g) vs. moderate LBW (2240–2499 g).

Variable	Odds Ratio	95% CI	*p*-Value	Interpretation
Gestational age	0.71	0.65–0.78	<0.001	Protective factor
Newborn height	0.41	0.36–0.47	<0.001	Strong protective factor
Hospitalization	0.85	0.77–0.94	0.003	Moderate protective effect
Maternal education	0.95	0.91–0.99	0.020	Mild protective effect
Prenatal visits	0.93	0.89–0.98	0.010	Protective factor
Maternal age	0.99	0.97–1.01	0.280	Not statistically significant
Epidemiological week	1.01	0.99–1.03	0.120	No strong evidence of effect

## Data Availability

The dataset analyzed in this study corresponds to microdata from Colombia’s SIVIGILA health surveillance system and was publicly available at https://medata.gov.co/dataset/1-026-22-000129 at the time of data collection (accessed on 15 February 2025). Should the link become temporarily inaccessible, the deidentified dataset is available upon reasonable request to the corresponding author (vmorales@correo.unicordoba.edu.co).

## Abbreviations

The following abbreviations are used in this manuscript:

LBW	Low Birth Weight
ELBW	Extreme Low Birth Weight (as defined in this study: birth weight < $Q_{1}$ = 2240 g)
MLBW	Moderate Low Birth Weight (as defined in this study: 2240–2499 g)
SIVIGILA	Sistema de Vigilancia en Salud Pública
SMOTE	Synthetic Minority Oversampling Technique
ADASYN	Adaptive Synthetic Sampling
GAMs	Generalized Additive Models
WHO	World Health Organization
AUC	Area Under the ROC Curve
OR	Odds Ratio
Spec	Specificity
Sens	Sensitivity
ACC	Accuracy

## Appendix A. Algorithmic Workflow for Extreme LBW Prediction

**Algorithm** **A1** Quantile-Based Logistic Modeling for Extreme Low Birth Weight Prediction
1: **Input:** SIVIGILA birth registry dataset *D* (LBW records only, *n* = 10,185) 2: **Data preprocessing** 3: Remove implausible birth weights (<500 g or >6000 g) 4: Remove records with missing or inconsistent values (>10% per variable) 5: Select predictors: gestational age, maternal age, education level, prenatal visits, hospitalization status, newborn height, epidemiological week 6: **Outcome redefinition** 7: Compute first quartile of birth weight $Q_{1}$ 8: Define outcome variable: $Y^{*}$ = 1 if BirthWeight < $Q_{1}$; $Y^{*}=0$ if $Q_{1}\leq$ BirthWeight < 2500 g 9: Standardize continuous predictors (zero mean, unit variance) 10: Split data: training set (70%), test set (30%), stratified by $Y^{*}$ 11: **Model estimation** 12: Fit five predictive models: (1) Traditional Logistic, (2) Balanced Logistic with Class Weights, (3) Logistic + SMOTE, (4) Penalized Elastic Net, (5) Penalized + SMOTE 13: Tune Elastic Net hyperparameters (α, λ) via 5-fold cross-validation 14: **Model evaluation on test set** 15: Compute AUC, accuracy, sensitivity, specificity, and F1 score 16: Evaluate calibration: Brier score and calibration curves 17: Extract logistic regression coefficients and compute odds ratios with 95% CI 18: **Output:** Predictive performance comparison across models and interpretable risk factors for extreme LBW
